# Supplementary material for: Toxicity of extracellular alpha-synuclein is independent of intracellular alpha-synuclein
Source: Sci Rep. 2022 Dec 19;12:21951. doi: 10.1038/s41598-022-25790-2 (PMC9763379; doi:10.1038/s41598-022-25790-2)
Supplement: Supplementary file 1 — Supplementary Information. [file 41598_2022_25790_MOESM1_ESM.docx]

**Toxicity of extracellular alpha-synuclein is independent of intracellular alpha-synuclein**

Yanina Dening ^1,2,*^, Theresa Straßl ^2,*^, Viktoria Ruf ^3,4^ Petra Dirscherl ^5^, Alexandra Chovsepian ^1^, Alicia Stievenard ^6^, Amit Khairnar ^7,#^, Felix Schmidt ^2,3^, Florian Giesert ^5^, Jochen Herms ^3,4,8^ , Johannes Levin ^2,4,8^, Marianne Dieterich ^2,4^, Peter Falkai ^1^, Daniela Vogt Weisenhorn ^5^ , Wolfgang Wurst ^5,8,9^ , Armin Giese ^3,4^ and Francisco Pan-Montojo^1,2,4,¥^

*^1^ Department of Psychiatry, Ludwig-Maximilian University Hospital, Nußbaumstr. 7, 80366, Munich*

*^2^ Department of Neurology, Ludwig-Maximilian University Hospital, Marchioninistr. 15, 81377 Munich*

*^3^ Center for Neuropathology und Prion Research, Ludwig-Maximilian-Universität München, Feodor-Lynen Str. 23, 81377 Munich*

*^4^ Munich Cluster for Systems Neurology, SyNergy*

*^5^ Institute of Developmental Genetics, Helmholtz Zentrum Munich, Germany*

*^6^ Univ. Lille, Inserm, CHU Lille, UMR-S 1172 - JPArc - Centre de Recherche Jean-Pierre AUBERT Neurosciences et Cancer, F-59000 Lille, France*

*^7^ Applied Neuroscience Research Group, CEITEC - Central European Institute of Technology, Masaryk University, Brno, Czech Republic*

*^8^ Deutsches Zentrum für Neurodegenerative Erkrankungen, Feodor-Lynen Str. 17, 81377 Munich*

*^9^ Technische Universität München-Weihenstephan 85764 Neuherberg/Munich, Germany*

^¥^ *Corresponding author:* [*francisco.pan-montojo@med.uni-muenchen.de*](mailto:francisco.pan-montojo@med.uni-muenchen.de)

^*^ *These authors contributed equally and are listed in alphabetical order*

*^#^ National Institute of Pharmaceutical Education and Research (NIPER), Ahmedabad, Palaj, Gandhinagar-382355, Gujarat, India*

**Supplementary Figure 1: Alpha-synuclein knock-out (ASYN KO) and Green Fluorescent-Protein (GFP) genotyping**

Picture of genotyping results for GFP+ (A) and ASYN KO (B) mice. A) **+**= positive control, **neg** = negative control (water). B) + = positive control (ASYN KO mice: 192 bp (mutant)), **het** = heterozygous mice: 105 bp (Wild-type) and 192 bp (mutant), neg = negative control (water). These results are in accordance to the expected results according to the provider.


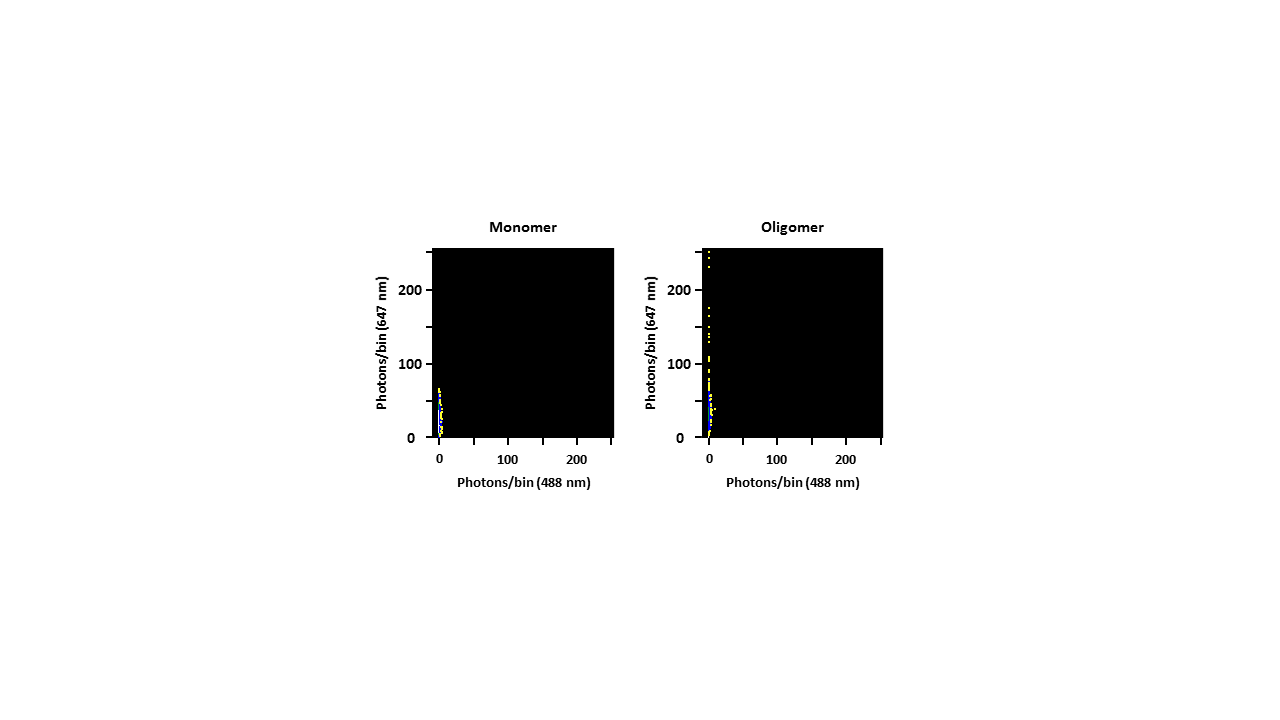


**Supplementary Figure 2:**  2D-FIDA Histogram showing the size distribution of Alexa Fluor-647 tagged ASYN incubated in the absence (left) or presence (right) of 100µM Al^3+^.


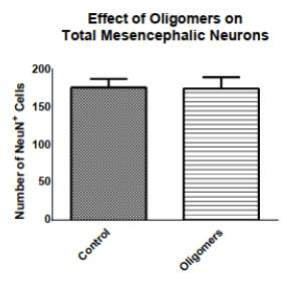
**Supplementary Figure 3: Effect of alpha-synuclein (ASYN) oligomers on the total amount of Neuronal nuclei-positive (NeuN+) neurons in mesencephalic cultures.**  Bar graph showing the total amount of NeuN+ neurons in control treated and ASYN- oligomers treated mesencephalic cultures. The number in the y-axis shows the number of cells quantified in a vertical line from top to bottom for each well. Error bars represent standard error of mean (SEM).
